# Uniaxial Stress Enhanced Anisotropic Magnetoresistance and Superconductivity in the Kagome Superconductor LaRu_3_Si_2_

**DOI:** 10.1002/adma.74067

**Published:** 2026-07-13

**Authors:** P. Kral, V. Sazgari, Y. Ge, O. Gerguri, M. Spitaler, J.N. Graham, H. Nakamura, M. Bartkowiak, S. Nakatsuji, H. Luetkens, G. Simutis, G. Xu, Z. Guguchia

**Affiliations:** ^1^ PSI Center for Neutron and Muon Sciences CNM Paul Scherrer Institut Switzerland; ^2^ Wuhan National High Magnetic Field Center and School of Physics Huazhong University of Science and Technology Wuhan China; ^3^ Institute for Solid State Physics (ISSP) University of Tokyo Chiba Japan

**Keywords:** anisotropy, condensed matter physics, critical field, density of states, electronic structure, ground state, magnetoresistance, physics, superconductivity

## Abstract

To elucidate how kagome electronic structure governs quantum ground states, we introduce a unique approach combining in‐plane uniaxial stress tuning, magnetotransport, and first‐principles calculations to uncover its impact on superconducting and normal‐state properties in ${\mathrm{LaRu}}_{3}{\mathrm{Si}}_{2}$. We identify a pronounced anisotropy in both the upper critical field and the normal‐state magnetoresistance, indicating strong electronic anisotropy despite the three‐dimensional crystal structure. Furthermore, we find that the superconducting transition temperature $T_{c}$ increases under in‐plane stress applied within the kagome plane, although the enhancement is modest, reaching approximately 0.3 K at 0.6 GPa. Furthermore, the absolute magnetoresistance exhibits a pronounced increase from about 22% at zero stress to 35% at 0.6 GPa, indicating a substantial modification of the normal state above $T_{c}$. The simultaneous enhancement of both $T_{c}$ and magnetoresistance under stress suggests a positive correlation between superconductivity and normal‐state electronic and magnetic properties in ${\mathrm{LaRu}}_{3}{\mathrm{Si}}_{2}$. Detailed calculations demonstrate that the stress‐induced evolution of superconductivity and magnetotransport arises from the cooperative interplay between modifications of the total density of states, kagome flat‐band physics, and anisotropic electronic response. In particular, the pronounced enhancement of magnetoresistance is closely linked to the stress‐driven downward shift of the Ru $dz^{2}$ kagome‐derived flat band.

## Graphical abstract

We identify strong anisotropy in the upper critical field and magnetoresistance in the kagome superconductor LaRu3Si2, revealing pronounced electronic anisotropy. Uniaxial stress enhances magnetoresistance and Tc, with a positive correlation suggesting an electronic pairing mechanism. The magnetoresistance increase originates from a stress‐driven downward shift of the Ru dz2 kagome flat band, demonstrating that uniaxial stress can tune flat bands and control both normal‐state and superconducting properties

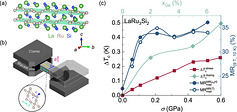

## 1 Introduction

Kagome superconductors have emerged as a fertile ground for investigating correlated electron behavior, owing to their geometrically frustrated lattice [1] and the delicate balance between competing quantum states [2, 3, 4, 5, 6, 7, 8, 9, 10, 11]. This competition – particularly between superconductivity and charge order – renders these systems highly tunable through external parameters such as chemical substitution or the application of mechanical pressure [12, 13, 14, 15, 16, 17].

Besides[Supplementary-material adma74067-supl-0001] the deeply studied family of *A* $V_{3}{\mathrm{Sb}}_{5}$ (*A* = K, Rb, Cs) [2, 3, 10, 12, 18] showing an exceptionally rich spectrum of electronic properties including unconventional superconductivity competing with charge order for the same electronic states, ${\mathrm{LaRu}}_{3}{\mathrm{Si}}_{2}$ [19, 20, 21, 22, 23, 24] represents an excellent opportunity to further extend the study of entanglement between these two phenomena. Featuring kagome superconductivity below $T_{c}$ ≈ 7 K and an unusually high charge‐ordering temperature ($T_{\mathrm{co},I}$ ≈ 400 K), followed by the emergence of a secondary charge order below $T_{\mathrm{co},\mathrm{II}}$ ≈ 80 K and a weak magnetic state below $T^{*}$ ≈ 35 K accompanied by large magnetoresistance, this system provides a unique platform for exploring the intricate interplay among intertwined quantum states in kagome materials. Related compounds [25, 26, 27] from the same structural family have recently been shown to exhibit similarly rich physics, thereby bringing the 132 family of kagome superconductors into greater prominence and making it an increasingly attractive platform for investigating correlated quantum phenomena.

Recent high‐pressure studies on ${\mathrm{LaRu}}_{3}{\mathrm{Si}}_{2}$ [17, 28] reveal a dome‐shaped superconducting phase diagram with an optimal transition temperature of $T_{c}\approx 9$ K, the highest reported amongst kagome superconductors. This evolution of $T_{c}$ appears to originate from an intricate interplay between superconductivity, charge order, and the underlying normal‐state electronic response. In particular, pressure uncovers a clear positive correlation between superconductivity and normal‐state electronic features associated with the characteristic temperature scale $T^{*}$. Moreover, pressure studies demonstrate a strong correlation between $T_{c}$ and the spatial coherence of the charge order: as long as the charge order remains long‐ranged, $T_{c}$ reaches its optimal value, whereas a pressure‐induced crossover from long‐ to short‐range charge order leads to a substantial suppression of $T_{c}$. These findings highlight the importance not only of the presence of charge order, but also of its spatial character and its coupling to the electronic and magnetic landscape of the normal state. In ${\mathrm{LaRu}}_{3}{\mathrm{Si}}_{2}$, the charge order is intimately linked to distortions within the Ru layer, from which time‐reversal‐symmetry (TRS) breaking also emerges [16, 20]. This strongly suggests that the kagome lattice plays a central role in governing superconductivity, charge order, and TRS breaking, providing a natural explanation for the observed direct correlation between superconductivity and charge order. A key next step is therefore to identify external tuning parameters that can optimize the Ru‐site distortions and the associated electronic responses to further enhance $T_{c}$. In this context, applying uniaxial stress along multiple crystallographic directions offers a particularly promising route to selectively tune Ru‐site distortions, $T^{*}$, and $T_{\mathrm{co},\mathrm{II}}$, with the potential to further increase the superconducting transition temperature. Uniaxial stress has been shown to be an efficient tool to tune the superconductivity and magnetism in cuprates [29, 30, 31, 32] and ${\mathrm{Sr}}_{2}{\mathrm{RuO}}_{4}$ [33, 34], however, the effect of uniaxial compression is much less explored in the family of kagome materials [13, 35, 36, 37].

**FIGURE 1:**
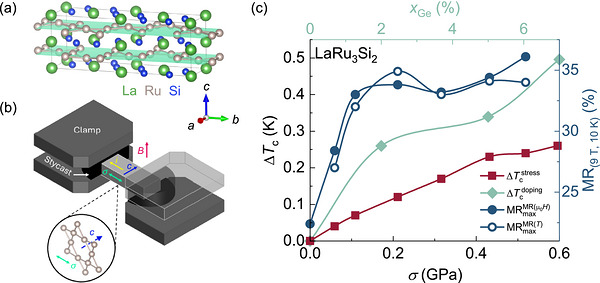
(a) ${\mathrm{LaRu}}_{3}{\mathrm{Si}}_{2}$ crystallographic unit cell with kagome planes subjected to the uniaxial stress, highlighted in green. (b) A simplified schematic illustration of the uniaxial stress setup, with the directions of the applied stress, transport current, and magnetic field indicated. The experimental configuration follows that shown in Figure 2c, focusing on the case in which the normal‐state response is already enhanced by the intrinsic anisotropy. (c) Uniaxial stress dependence of the superconducting transition temperature (left axis) and the magnetoresistance (right axis) in ${\mathrm{LaRu}}_{3}{\mathrm{Si}}_{2}$. The chemical doping (${\mathrm{LaRu}}_{3}$(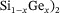) effect on $T_{c}$ is shown for comparison, as well [38].

In this work, we first investigate the anisotropic electronic response of ${\mathrm{LaRu}}_{3}{\mathrm{Si}}_{2}$ using detailed magnetotransport measurements. By systematically varying the orientations of the electrical current and magnetic field with respect to the crystallographic c‐axis, we reveal a pronounced directional dependence in both the normal and superconducting states. Building on these results, we then explore the effects of in‐plane uniaxial stress, focusing on the configuration in which the intrinsic anisotropy already enhances the normal‐state response. The key results of our uniaxial‐stress experiments together with the schematic illustration of the experimental setup are summarized in Figure 1. Upon applying in‐plane uniaxial stress of up to 0.6 GPa, we observe a modest increase in the superconducting transition temperature $T_{c}$ accompanied by a substantial enhancement of the magnetoresistance, establishing a positive correlation between the two quantities. The strong increase in magnetoresistance originates from a stress‐driven downward shift of the Ru $dz^{2}$ kagome flat band. As established previously, the magnetoresistance tracks the onset of charge order and TRS breaking. This positive correlation indicates that the TRS‐breaking normal state does not compete with, but rather enhances, superconductivity–pointing to a cooperative interplay in which the normal‐state electronic (magnetic) response promotes superconducting pairing and supports an electronically driven mechanism. Together, these results link flat‐band tuning to both normal‐state transport and superconductivity, revealing a unified framework governed by flat‐band physics.

## 2 Results and Discussion

### 2.1 Stress‐Tuned Superconducting and Magnetotransport Responses

**FIGURE 2:**
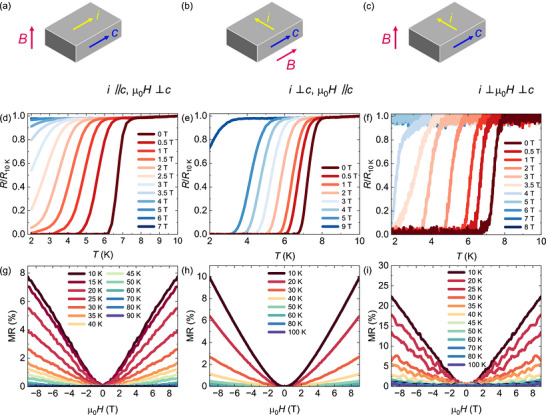
(a–c) Schematic representations of the experimental configurations with varying orientations of the electrical current and magnetic field relative to the crystallographic c‐axis. Panels below display the corresponding measurements for each configuration. The uniaxial‐stress experiment was performed in configuration (c). (d–f) Electrical resistivity across the superconducting transition measured under the application of various magnetic fields. (g–i) Magnetoresistance curves measured at selected temperatures for each of the three measurement configurations.

Figure 2 summarizes the magnetotransport measurements of ${\mathrm{LaRu}}_{3}{\mathrm{Si}}_{2}$ in both the superconducting and normal states, obtained for different orientations of the electrical current and magnetic field with respect to the crystallographic c‐axis. The three experimental configurations investigated are illustrated in Figure 2a–c. The temperature dependence of the resistivity across the superconducting transition for these configurations is shown in Figure 2d–f. Well‐defined superconducting transitions are observed in all cases, with a midpoint transition temperature of approximately 7 K. Notably, the upper critical field $\mu_{0}H_{c2}$ exhibits a pronounced anisotropy, varying significantly among the three configurations. For the two configurations with the magnetic field in the Kagome plane, i∥c, $\mu_{0}H⊥c$ and $i⊥\mu_{0}H⊥c$, investigated in the present work, the upper critical field is nearly identical, with $\mu_{0}H_{c2}$ ≃ 4–5 T at 2 K. A slightly higher value of 5 T has been previously reported for the configuration $i∥\mu_{0}H∥c$ [20]. In contrast, for the configuration i⊥c, $\mu_{0}H∥c$, the superconductivity is significantly more robust, with the upper critical field exceeding $\mu_{0}H_{c2}$ > 9 T at 2 K. These results demonstrate that the relative orientation of the electrical current and magnetic field plays a decisive role in determining the magnitude of $\mu_{0}H_{c2}$. The upper critical field $\mu_{0}H_{c2}$ is governed by Cooper‐pair breaking mechanisms, which can be classified as either orbital pair breaking–arising from vortex formation due to the Lorentz force–or Pauli (Zeeman) pair breaking, originating from spin polarization. Orbital pair breaking is inherently anisotropic and depends sensitively on the orientation of the applied magnetic field relative to the crystallographic axes and the Fermi‐surface geometry, whereas Pauli limiting is largely isotropic unless strong spin–orbit coupling is present. For magnetic fields applied parallel to the crystallographic c‐axis (H∥c), vortices penetrate perpendicular to the kagome planes. In this configuration, the relevant orbital length scale is set by the in‐plane superconducting coherence length. A higher upper critical field $\mu_{0}H_{c2}$ along the c‐axis implies that superconductivity is more robust against orbital pair breaking for vortices aligned along c, which requires a shorter in‐plane coherence length (tighter vortex cores in the plane). Short coherence length is also indicative of spatially compact Cooper pairs and a strongly coupled superconducting state. This behavior is naturally consistent with reduced Fermi velocity and enhanced electronic correlations. Within this framework, the pronounced anisotropy of both $\mu_{0}H_{c2}$ and MR reflects an underlying electronic anisotropy in ${\mathrm{LaRu}}_{3}{\mathrm{Si}}_{2}$, despite the three‐dimensional crystal structure, rooted in kagome‐derived electronic states, which set the anisotropic orbital pair breaking. Moreover, the data indicate that superconductivity in ${\mathrm{LaRu}}_{3}{\mathrm{Si}}_{2}$ is predominantly orbital‐limited rather than Pauli‐limited.

To further elucidate the anisotropic behavior, we focus on the normal‐state response that is closely linked to superconductivity [29]. In particular the magnetoresistance, which reflects the onset of secondary charge‐order formation in ${\mathrm{LaRu}}_{3}{\mathrm{Si}}_{2}$ below $T_{\mathrm{co},\mathrm{II}}$ ≈ 80 K and the emergence of a weak magnetic state below $T^{*}$ ≈ 35 K. Magnetoresistance curves measured at selected temperatures are shown in Figure 2g–i. Independent of the measurement configuration, the magnetoresistance onset is observed below 80 K, followed by a pronounced enhancement at temperatures below 35 K. Notably, the relative orientation of the magnetic field and electrical current has a strong impact on the magnitude of the magnetoresistance. In the configurations i∥c, $\mu_{0}H⊥c$, and $i∥\mu_{0}H∥c$ (reported previously), a moderate magnetoresistance of approximately 8 % is observed at T = 10 K and $\mu_{0}H$ = 9 T. For the configuration i⊥c, $\mu_{0}H∥c$, the magnetoresistance increases to about 10 %. In contrast, for $i⊥\mu_{0}H⊥c$, the magnetoresistance is strongly enhanced, reaching nearly 23 %, exceeding the maximum value of 14 % reported under hydrostatic pressure [29]. These results, summarized in Table 1, reveal a pronounced anisotropy of both superconductivity and normal‐state magnetoresistance in ${\mathrm{LaRu}}_{3}{\mathrm{Si}}_{2}$, pointing to a strong intertwining of their underlying physics.

**TABLE 1:** Summary of the anisotropic superconducting and normal‐state properties of ${\mathrm{LaRu}}_{3}{\mathrm{Si}}_{2}$: the configuration‐dependent upper critical field $\mu_{0}H_{c2}$ (2 K) and maximum magnetoresistance ${\mathrm{MR}}_{\max}$ (9 T, 10 K). The uniaxial‐stress experiment is performed in configuration (c) with the maximum normal‐state response.

Configuration	$\mu_{0}H_{c2}$ (T)	${\mathrm{MR}}_{\max}$ (%)
(a) i∥c, $\mu_{0}H⊥c$	≃ 4–5 T	8
(b) i⊥c, $\mu_{0}H∥c$	> 9 T	10
(c) i⊥c, $\mu_{0}H⊥c$	≃ 4–5 T	23

**FIGURE 3:**
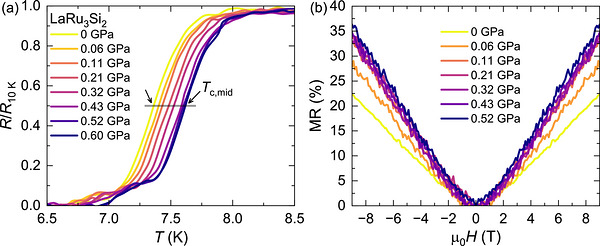
(a) The temperature dependence of electrical resistivity, normalized to its value at 10 K, for ${\mathrm{LaRu}}_{3}{\mathrm{Si}}_{2}$, measured under various in‐plane uniaxial stress values. (b) The field dependence of magnetoresistance, measured under various in‐plane uniaxial stress values. These measurements were performed with the following configuration $i⊥\mu_{0}H⊥c$ (Figure 2c).

Focusing on the configuration with the strongest magnetotransport response (see Figure 1a), we further aim to explore the subtle interplay between superconductivity and the normal‐state electronic properties through in‐plane uniaxial stress. Figure 3a depicts the stress effect on the superconducting transition, clearly indicating the gradual increase in all the onset, midpoint, and zero‐resistance characteristic temperatures setting the in‐plane uniaxial stress as an effective tool for optimizing the superconducting properties. The stress‐driven increase in $T_{c}$ is accompanied by the significant enhancement of the maximum value of magnetoresistance reached, from ambient ≈23% to the saturation at ≈35% above σ≈ 0.1 GPa. Figure 1b summarizes the evolution of the superconducting transition temperature and the maximum magnetoresistance (measured at T = 10 K and $\mu_{0}H$ = 9 T) as a function of uniaxial stress. The data show a gradual increase in $T_{c}$ with increasing stress, reflecting a clear enhancement of the superconducting properties. The magnetoresistance follows a similar overall trend, however, initially rising rather sharply before eventually saturating at the maximum value of ≈35% at higher stress applied. While stress‐induced modifications of charge‐order domains could, in principle, influence MR, our results point to an intrinsic electronic origin rather than domain‐wall contributions. In particular, the onset of MR coincides with a sign change in the Hall effect [20], indicating a Fermi surface reconstruction. Moreover, the emergence of TRS breaking [20] at a similar temperature scale further supports its unconventional, intrinsic nature. The strongly anisotropic MR observed here is also difficult to reconcile with a domain‐wall scenario. Instead, as we show below, the pronounced stress dependence of MR is naturally explained by a stress‐driven downward shift of the Ru $dz^{2}$ kagome flat band.

The simultaneous enhancement of both $T_{c}$ and magnetoresistance under in‐plane uniaxial stress constitutes a key finding of this work. The positive stress dependence of $T_{c}$ is encouraging, as it motivates extending uniaxial stress to higher values with the potential for further enhancement of the superconducting transition temperature. More strikingly, the magnetoresistance increases by approximately 60% under a relatively small in‐plane uniaxial stress of only 0.1 GPa. As discussed above, the onset of magnetoresistance coincides with the emergence of secondary charge order, while its strongest enhancement occurs below 35 K, the temperature at which muon‐spin rotation measurements reveal the onset of weak magnetism. The pronounced stress‐induced enhancement of MR therefore points to a modification of the charge‐ordered state and the associated electronic structure, an enhancement of the magnetic response, or a combined effect of both. The strong sensitivity of the normal‐state electronic and magnetic properties to in‐plane uniaxial stress makes ${\mathrm{LaRu}}_{3}{\mathrm{Si}}_{2}$ particularly intriguing from both fundamental and potential application perspectives. Furthermore, the observed positive correlation between $T_{c}$ and magnetoresistance highlights a strong interdependence between superconductivity and normal‐state electronic or magnetic properties, suggesting a common underlying mechanism. Taken together, these results strongly support an electronically driven superconducting state in ${\mathrm{LaRu}}_{3}{\mathrm{Si}}_{2}$.

### 2.2 Stress‐Tuned Electronic Structure From First‐Principles Calculations

**FIGURE 4:**
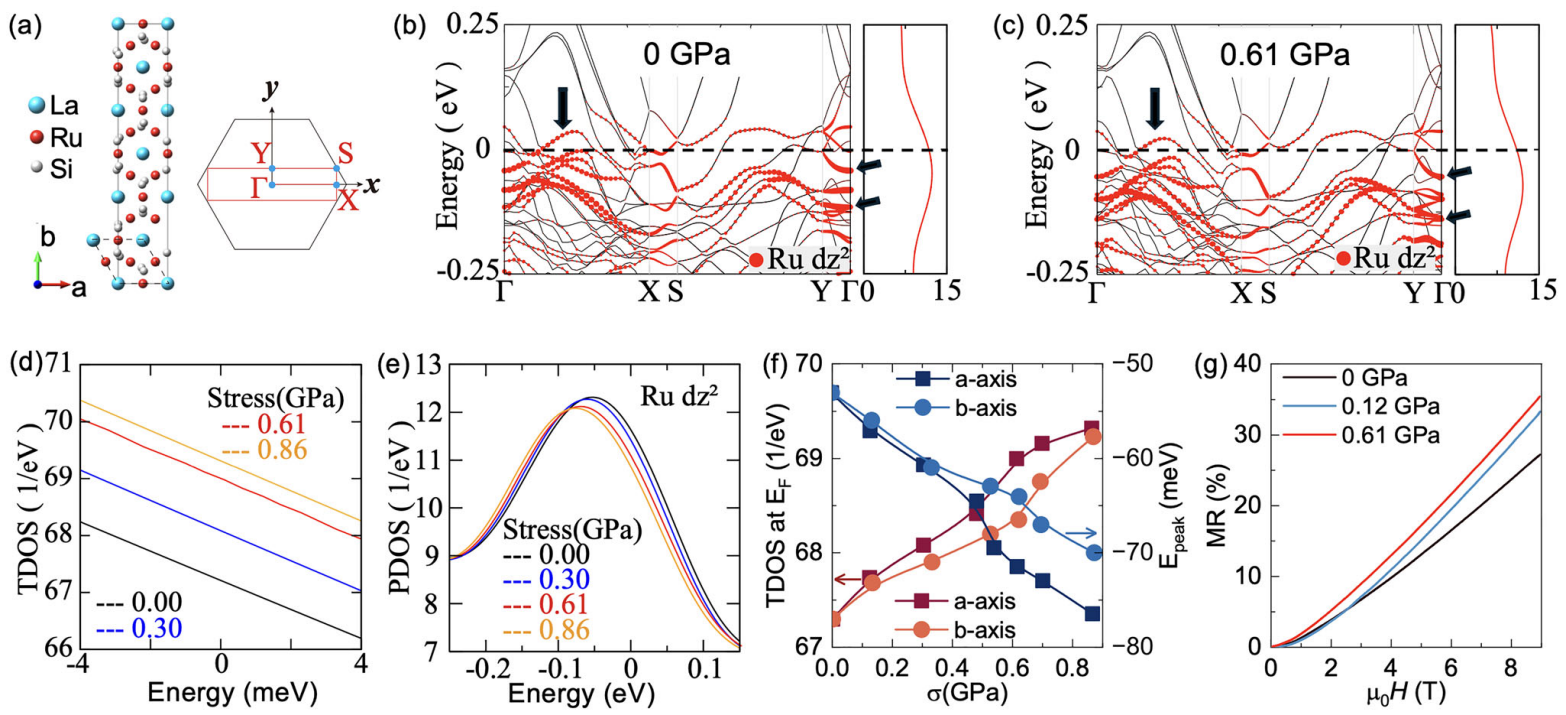
(a) Crystal structure and Brillouin zone ($k_{z}=0$ plane) of the CO‐II phase, with the folded Brillouin zone induced by structural distortion indicated. (b,c) Band structures and projected density of states (PDOS) at 0 GPa (b) and under 0.61 GPa uniaxial stress along the a‐axis (c). The size of the red circles scales with the spectral weight of the Ru $d_{z^{2}}$ orbitals. Dashed lines and arrows are intended to guide the reader in identifying stress‐induced changes. The dashed lines indicate the Fermi level, while the arrows highlight regions showing a continuous shift of the bands away from the Fermi level. (d) Evolution of the total density of states (TDOS) near the Fermi level under uniaxial stress along the a‐axis. (e) Evolution of the Ru $d_{z^{2}}$ PDOS as a function of uniaxial stress. (f) (left axis) Calculated TDOS at the Fermi level as a function of uniaxial stress applied along the a and b axes. (right axis) Energy position of the Ru $d_{z^{2}}$‐dominated flat‐band peak center as a function of uniaxial stress along the a and b axes. (g) Calculated magnetoresistance as a function of magnetic field H for different a‐axis uniaxial stress values.

To elucidate the microscopic origin of the uniaxial‐stress effects, we performed first‐principles calculations as a function of applied stress. The intrinsic structural distortion of the Ru kagome sublattice leads to a reconstruction of the Brillouin zone (BZ), as illustrated in Figure 4a. The calculated band structures and projected density of states (PDOS) of the Ru $d_{z^{2}}$ orbitals at 0 GPa and under 0.61 GPa uniaxial stress applied along the *a*‐axis are shown in Figure 4b,c, respectively. As shown in Figure 4b, some quasi‐flat bands close to the Fermi level are observed in the whole reconstructed BZ. These bands are dominated by Ru $d_{z^{2}}$ orbitals and obtained by the folding effect of kagome flat band for the SG 193 structure in previous reports [20]. From these calculations, we extracted several key quantities: (i) the evolution of the total density of states (TDOS) near the Fermi level under uniaxial stress (Figure 4d); (ii) the evolution of the Ru $d_{z^{2}}$ flat bands and PDOS under uniaxial stress (Figure 4e) (the detailed results and analysis can be found in Supplementary Information);(iii) the calculated magnetoresistance as a function of magnetic field and a‐axis uniaxial stress (Figure 4g). With increasing *a*‐axis uniaxial stress, the electronic structure evolves with two characteristic trends (Figure 4f): a monotonic increase in the total TDOS (Figure 4f left axis) and a concomitant downward shift of the Ru $d_{z^{2}}$ quasi‐flat band in energy (Figure 4f right axis). A qualitatively similar behavior is found for stress applied along the *b*‐axis.

In the following, we discuss how the theoretical results align with the experimental observations, namely a modest stress‐induced enhancement of $T_{c}$ and a pronounced increase in magnetoresistance. The calculated enhancement of the TDOS at the Fermi level (Figure 4d,f) can contribute to the observed increase in $T_{c}$. In contrast, the stress‐induced shift of the Ru $d_{z^{2}}$ PDOS peak (Figure 4e,f) indicates that the kagome‐derived flat band moves away from $E_{F}$. This shift is expected to weaken electronic correlations and reduce the flat‐band contribution to superconducting pairing, which would otherwise favor a higher $T_{c}$. As a consequence, two competing effects are at play: the increase in TDOS tends to enhance $T_{c}$, while the reduction of flat‐band correlations counteracts this tendency. The resulting modest net increase in $T_{c}$ therefore likely reflects the displacement of the flat band away from the Fermi level, which limits the overall enhancement of superconductivity. Importantly, the same stress‐induced shift of the flat band has a much stronger impact on the normal‐state transport. As the flat band moves away from $E_{F}$, the heavy carriers' contribution to the Fermi surface is reduced, leading to a decrease in the average quasiparticle effective mass $m^{*}$. Within the Chambers transport formalism  [39], a reduction in the effective mass enhances the cyclotron frequency, resulting in more rapid deflection of carriers and thereby tending to enhance the magnetoresistance. To validate this physical picture, we calculated the MR using a tight‐binding model based on SG 193 structure, since it shares similar distorted Ru‐kagome layers with the CO‐II phase and exhibits a consistent pressure‐driven evolution of the TDOS and PDOS. Our calculations (Figure 4g) successfully reproduce the experimentally observed large enhancement of the magnetoresistance. Furthermore, the calculations reveal a pronounced initial increase of the magnetoresistance (MR) between 0 and 0.12 GPa, followed by a much weaker enhancement at higher applied stress. This behavior is in excellent agreement with the experimental observations, further substantiating the consistency between experiment and theory. These results demonstrate that, while the stress‐induced change in $T_{c}$ arises from a combined evolution of the TDOS and the flat band, the dramatic increase in magnetoresistance can be explained predominantly–if not entirely–by the stress‐driven evolution of the kagome flat band.

An increase of $T_{c}$ in ${\mathrm{LaRu}}_{3}{\mathrm{Si}}_{2}$ upon substituting Si with Ge has been reported recently [28]. This behavior contrasts sharply with chemical substitution at the Ru site, which generally leads to a pronounced suppression of superconductivity [23, 40], underscoring a fundamental difference between the substitution mechanisms. Replacing Si with the larger Ge atom induces a strongly anisotropic lattice expansion, dominated by an elongation along the crystallographic c‐axis. This response is analogous to the effect of in‐plane uniaxial stress, which similarly induces a c‐axis expansion. However, Ge substitution may introduce chemical disorder, complicating the disentanglement of disorder and lattice effects. In contrast, the mechanical tuning employed here provides a cleaner route that preserves the intrinsic chemical structure, enabling a direct assessment of c‐axis effects on the electronic structure, superconductivity, and magnetoresistance. This constitutes a key advantage of our approach and provides access to the intrinsic stress response, offering unique insight into the underlying physics.

## 3 Conclusion and Outlook

In conclusion, we combine magnetotransport measurements and density‐functional‐theory (DFT) calculations to investigate the superconducting and normal‐state properties of ${\mathrm{LaRu}}_{3}{\mathrm{Si}}_{2}$ under in‐plane uniaxial stress. We first identify a pronounced intrinsic anisotropy in both superconducting and normal‐state responses, with the upper critical field and the magnitude of the normal state magnetoresistance strongly dependent on the relative orientations of the electrical current and magnetic field with respect to the crystallographic c‐axis. The magnetoresistance reaches its maximum when both the electrical current and the magnetic field are oriented perpendicular to the c‐axis. The strong dependence of the upper critical field and magnetoresistance on magnetic‐field orientation, despite identical current directions, demonstrates that superconductivity in ${\mathrm{LaRu}}_{3}{\mathrm{Si}}_{2}$ is governed by anisotropic orbital pair breaking. Given that the dominant electronic states near the Fermi level arise from kagome layers (Ru d‐orbitals), and that our first‐principles calculations reveal pronounced flat‐band features and dispersion anisotropy, we attribute the observed anisotropy to the kagome‐derived electronic structure, which governs the orbital pair breaking. Focusing on the configuration, which maximizes the normal‐state MR response, we apply in‐plane uniaxial stress up to 0.6 GPa and observe a monotonic increase of the superconducting transition temperature $T_{c}$. Although the enhancement of $T_{c}$ is modest, the positive pressure dependence is notable. In contrast, the magnetoresistance exhibits a substantial enhancement of up to 60% under in‐plane compression. Our calculations indicate that the stress‐induced change in $T_{c}$ reflects the combined evolution of the TDOS and the flat band, while the large magnetoresistance enhancement is dominated by the stress‐driven evolution of the kagome flat band. Given that the onset of weak magnetism coincides with the emergence of magnetoresistance, these results further suggest a stress‐induced modification of the magnetic response. Taken together, our findings reveal a positive coupling between superconductivity and normal‐state electronic properties, highlight the central role of the kagome flat band in both the normal and superconducting states, and point to an electronically driven superconducting state in ${\mathrm{LaRu}}_{3}{\mathrm{Si}}_{2}$.

The present results establish uniaxial stress as a powerful and selective tuning parameter for flat‐band systems, enabling direct control over both normal‐state electronic responses and superconducting properties. This approach opens a new route to engineer correlated quantum states by manipulating flat‐band dispersions in situ. Given the central role of flat bands across a wide range of materials–including kagome lattices, pyrochlores, heavy‐fermion systems, and moirè platforms such as graphene–our findings provide a broadly applicable framework for exploring and controlling emergent phenomena. Looking forward, combining strain with complementary tuning knobs such as pressure, magnetic field, and disorder, as well as extending these studies to higher fields and lower temperatures, may reveal new regimes of electronic order and help establish universal connections between flat‐band physics and unconventional superconductivity.

## 4 Methods

### 4.1 Uniaxial Stress Cell

For the uniaxial stress experiment, we employed the FC100 stress cell (Razorbill Instruments) compatible with the Physical Property Measurement System (PPMS, Quantum Design). A single‐crystalline ${\mathrm{LaRu}}_{3}{\mathrm{Si}}_{2}$ sample was mounted between two piezoelectrically actuated plates using Stycast epoxy. Magnetotransport measurements were performed using the standard four‐probe technique. The experimental geometry was configured according to Figure 2c, i.e., $i⊥\mu_{0}H⊥c$, with the stress direction parallel to the electrical current.

Razorbill FC100 is a force‐controlled stress cell, allowing direct measurement of the force applied on the sample. Knowing the sample width and thickness (0.9 and 0.4 mm, in this case), the corresponding stress can be determined. Within the experiment performed, the maximum force of 214 N was applied, corresponding to the stress of 0.60 GPa.

### 4.2 Computational Method

The first‐principles calculations were performed using the Vienna Ab initio Simulation Package (VASP) based on the density functional theory (DFT)  [41, 42, 43]. The exchange‐correlation functional was treated using the generalized gradient approximation (GGA) parameterized by Perdew, Burke, and Ernzerhof (PBE)  [44]. The cutoff energy for wave function expansion is 380 eV, and a 9×3×7 Monkhorst–Pack k‐point mesh was employed for the Brillouin zone sampling. To simulate the effects of in‐plane uniaxial stress, the CO‐II phase of ${\mathrm{LaRu}}_{3}{\mathrm{Si}}_{2}$ (SG 55) was optimized with the lattice parameter constrained along the applied stress direction, while allowing the remaining lattice parameters and all internal atomic positions to fully relax until the residual forces on each atom were less than 0.01 eV/Å. For the transport calculations, a tight‐binding Hamiltonian based on the room‐temperature structure (SG 193) was constructed using maximally localized Wannier functions (MLWFs) via the Wannier90 package  [45, 46, 47, 48]. Subsequently, magnetoresistance calculations were performed using the WannierTools package  [49, 50], taking into account the flat‐band‐induced quasiparticle mass renormalization.

## Author Contributions

Z.G. conceived, designed and supervised the project. Sample growth: H.K. and S.N¨ Magnetotransport experiments as a function of uniaxial stress: Z.G., P.K., V.S., O.G., M.S., J.N.G. with contribution from M.B¨ DFT calculations: Y.G. and G.X. in consultation with Z.G. Figure development and writing of the paper: Z.G. and P.K¨ All authors discussed the results, interpretation, and conclusion.

## Conflicts of Interest

The authors declare no conflicts of interest.

## Supporting information


**Supporting File**: adma74067‐sup‐0001‐SuppMat.pdf.

## Data Availability

The data that support the findings of this study are available from the corresponding authors upon request.
