# Supplementary material for: Uniaxial Stress Enhanced Anisotropic Magnetoresistance and Superconductivity in the Kagome Superconductor LaRu3Si2
Source: Adv Mater. 2026 Jul 13;38(46):e74067. doi: 10.1002/adma.74067 (PMC13486226; doi:10.1002/adma.74067)
Supplement: Supplementary file 1 [file ADMA-38-e74067-s001.pdf]

Uniaxial stress enhanced anisotropic magnetoresistance and superconductivity in the kagome superconductor $LaRu_3Si_2$

P. Král,[1,*] V. Sazgari,[1,*] Yongheng Ge,[2,*] O. Gerguri,[1] M. Spitaler,[1] J.N. Graham,[1] H. Nakamura,[3] M. Bartkowiak,[1] S. Nakatsuji,[3] H. Luetkens,[1] G. Simutis,[1] Gang Xu,[2,†] and Z. Guguchia[1,‡]

[1] *PSI Center for Neutron and Muon Sciences CNM, 5232 Villigen PSI, Switzerland*
[2] *Wuhan National High Magnetic Field Center and School of Physics, Huazhong University of Science and Technology, Wuhan 430074, China*
[3] *Institute for Solid State Physics (ISSP), University of Tokyo, Kashiwa, Chiba 277-8581, Japan*

(Dated: May 25, 2026)

Supplementary Note 1:

Since the effects of uniaxial pressure applied along the b-axis are qualitatively similar to those along the *a*-axis, we take the 0.61 GPa *a*-axis uniaxial pressure as a representative case, compared with the 0 GPa structure. The main changed structure parameters as illustrated in Figures S1(a) and S1(b) for both cases are summarized in Table 1. In summary, the pressure-induced changes can be summarized as follows:

First, because the uniaxial pressure does not change the crystal symmetry, the system remains in space group SG 55 both before and after the application of pressure.

Second, the applied pressure mainly induces small variations in bond lengths and bond angles. As shown in Table 1, after full structural relaxation, the in-plane bond length d_1 decreases by 0.01 , while the interlayer distance d_c increases by 0.03, reflecting the Poisson-effect response to in-plane uniaxial compression.

Third, as a result of these structural modifications, the interlayer hopping of d_{z2} is weakened, leading to the downshift of the d_{z2} antibonding states at the Fermi level as shown in Fig. S1(c). In contrast, the in-plane hopping of the d_{xy} and d_{x2-y2} orbitals is enhanced a little, causing the corresponding antibonding states near the Fermi level to shift toward higher energies. These two effects combined together give rise to a reasonable explanation of the enhancement of total DOS and the reduction of the d_{z2} DOS at the fermi level.

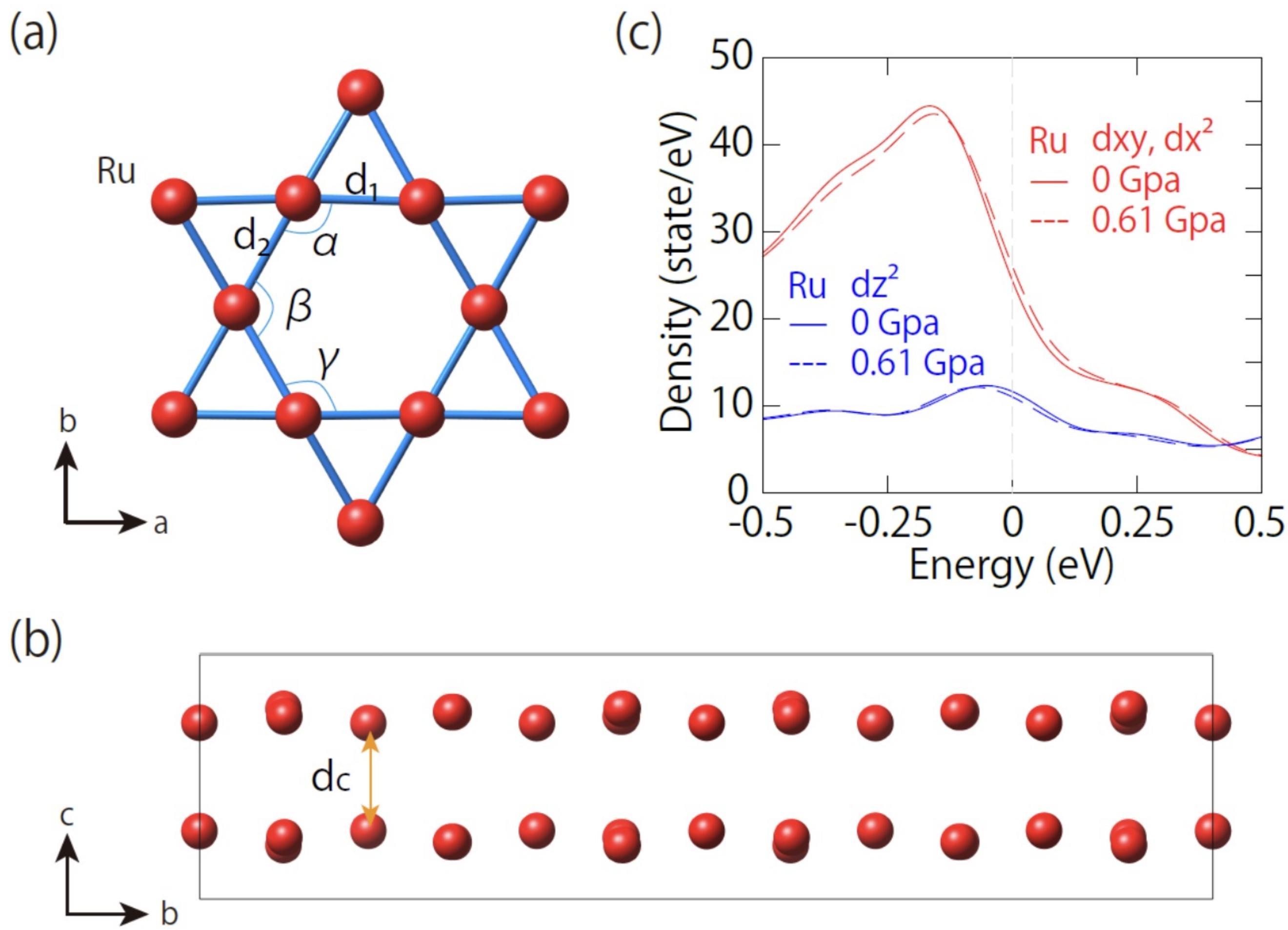

FIG. S1. Schematic illustration of the Ru kagome layer in the ab -plane (a) and bc-plane (b) for the CO-II phase of $LaRu_3Si_2$, showing the definitions of the in-plane bond lengths d_1 and d_2, the interlayer distance d_c, and the bond angles α, β and γ. (c) Calculated projected density of states for the Ru d_{xy}, d_{x2-y2}, and the d_{z2} at 0 GPa and under 0.61 GPa a-axis uniaxial stress.

Stress	$\alpha(^\circ)$	$\beta(^\circ)$	$\gamma(^\circ)$	d_1(Å)	d_2(Å)	d_c(Å)
0 GPa	118.08	119.09	117.75	2.86	2.89	3.15
0.61 GPa(a-axis)	118.23	119.3	116.95	2.85	2.89	3.18

TABLE S1. The structural parameters defined in Figure S1 under 0 GPa and 0.61 GPa a-axis uniaxial stress.
